# Marine Macroalgae Polyphenols as Potential Neuroprotective Antioxidants in Neurodegenerative Diseases

**DOI:** 10.3390/md21050261

**Published:** 2023-04-23

**Authors:** Silvia Lomartire, Ana M. M. Gonçalves

**Affiliations:** 1 University of Coimbra, MARE-Marine and Environmental Sciences Centre/ARNET-Aquatic Research Network, Department of Life Sciences, Calçada Martim de Freitas, 3000-456 Coimbra, Portugal; silvia.lomartire@student.uc.pt; 2 Department of Biology and CESAM, University of Aveiro, 3810-193 Aveiro, Portugal

**Keywords:** marine polyphenols, seaweeds, antioxidant activity, neurodegenerative diseases, neuroprotective activity

## Abstract

Polyphenols are beneficial natural compounds with antioxidant properties that have recently gain a lot of interest for their potential therapeutic applications. Marine polyphenols derived from marine macroalgae have been discovered to possess interesting antioxidant properties; therefore, these compounds can be included in several areas of drug development. Authors have considered the use of polyphenol extracts from seaweeds as neuroprotective antioxidants in neurodegenerative diseases. Marine polyphenols may slow the progression and limit neuronal cell loss due to their antioxidant activity; therefore, the use of these natural compounds would improve the quality of life for patients affected with neurodegenerative diseases. Marine polyphenols have distinct characteristics and potential. Among seaweeds, brown algae are the main sources of polyphenols, and present the highest antioxidant activity in comparison to red algae and green algae. The present paper collects the most recent in vitro and in vivo evidence from investigations regarding polyphenols extracted from seaweeds that exhibit neuroprotective antioxidant activity. Throughout the review, oxidative stress in neurodegeneration and the mechanism of action of marine polyphenol antioxidant activity are discussed to evidence the potential of algal polyphenols for future use in drug development to delay cell loss in patients with neurodegenerative disorders.

## 1. Introduction

The advantages of marine macroalgae (or seaweeds) to human wellbeing are well known [1,2,3,4]. Numerous bioactive molecules found in seaweeds may have health advantages against a range of diseases and conditions, including cancer, inflammation, microbes, and viruses [5,6,7,8,9,10,11,12]. The potential of seaweed bioactive compounds to act as a natural resource with remarkable neuroprotective properties can be based on abundant published results from recent clinical and preclinical studies. Numerous studies have documented seaweed bioactive compounds exhibiting therapeutic activities [13,14,15,16,17,18,19].

Seaweed biomass is a promising, renewable, and cost-effective [20,21,22] resource of high-value bioactive compounds that have been highly invested in within the food, pharmaceutical, and cosmetic industries [23,24,25,26,27,28,29].

Marine polyphenols have been discovered to be powerful antioxidant compounds; therefore, they can play a crucial role in the development of natural and innovative neuroprotective drugs.

Neuroprotection refers to methods and mechanisms that protect neuronal cells against injury, dysfunction, deterioration, and cell death in the central nervous system (CNS) [30]. These compounds may slow the progression and limit neuronal cell loss; therefore, the use of those would improve quality of life for patients affected with neurodegenerative diseases.

The progressive loss of specifically vulnerable groups of neurons characterizes neurodegenerative disorders, which are frequently (though not always) accompanied by neurodegenerative symptoms. Neurodegenerative diseases can be categorized according to their primary clinical characteristics, such as dementia, parkinsonism, or motor neuron disease; anatomical distribution of the disease, such as frontotemporal degenerations, extrapyramidal disorders, or spinocerebellar degenerations; or principal molecular abnormality [31]. Although the exact pathophysiology of neurodegenerative diseases is still unclear, common factors contribute to the disease progression: increased oxidative stress, neuroinflammation, misfolded proteins, dysfunctional mitochondria, and impaired proteostasis [32].

By interfering with the CNS functions, neurodegeneration affects both the structure and survival of neurons as well as their ability to operate. Neuronal cells of the CNS cannot grow back after being harmed by disease, ischemia (lack of oxygen, glucose, or blood flow), or physical trauma, in contrast to primary cells from the skin, liver, or muscle. The human CNS is incredibly complex, creating difficulties in the understanding and treatment of neurodegenerative diseases. Currently, no therapeutic treatments can stop the natural course of neurodegenerative disease, and treatments can only ameliorate the quality of life of patients affected by neurodegenerative diseases. Alzheimer’s disease (AD), frontotemporal lobar dementia (FTLD), Parkinson’s disease (PD), and amyotrophic lateral sclerosis (ALS) are some of the most common neurodegenerative diseases [33].

According to the research, exogenous antioxidants may benefit individuals with neurodegenerative diseases; it has been demonstrated that abundant biomolecules from marine sources show therapeutic potential. Phlorotannins, sulphated polysaccharides, carotenoids, and sterols are just a few of the powerful antioxidant compounds that have been found in various seaweeds. These marine organisms are valuable sources of compounds with neuroprotective effects, as they remove or suppress the generation of reactive oxygen species (ROS) and/or reactive nitrogen species (RNS), preventing neuronal cell death [34,35,36,37]. Emerging evidence suggests that antioxidant activity cannot be the exclusive mechanism by which compounds exert neuroprotection, and compounds that alter signalling pathways involved in cell survival systems have also been indicated [38].

Polyphenolic antioxidants have significant potential in free radical scavenging, which is a major contributor to neuronal damage and can therefore exert consequential neuroprotective effects and play a critical role in the treatment of neurodegenerative diseases.

Many studies have demonstrated the neuroprotective activity of polyphenolic antioxidants and their usefulness for neuronal regeneration. For example, in 2019, Zhou et al. [39] investigated the effect of luteolin, a natural flavonoid, on neurogenesis in Ts65Dn mice, a model of Down syndrome. The study found that luteolin significantly improved the behavioral performance of the mice, likely through promotion of neuronal differentiation and commutation in hippocampal neurogenesis. Similarly, Katebi et al. [40] introduced a new approach to neuronal repair therapeutics by combining quercetin and NGF with superparamagnetic iron oxide nanoparticles to efficiently promote neuronal branching in morphogenesis of PC12 cells. Bieler et al. [41] also investigated the effects of a prenylflavonoid on the regeneration of rat dorsal root ganglion neurons and found that enhancement of ENDF1, which was segregated from hops, could promote branching and induce a percentage of sensory neurons to regrow their neurites. In addition, various studies have shown positive neuroprotective effects of polyphenolic compounds such as myricetin, quercetin, tannic acid, and naringenin on both in vivo and in vitro experiments [42,43,44,45,46]. On this basis, the neuroprotective effects of seaweed compounds in various in vitro and in vivo models of neurodegeneration are further discussed in the present review.

## 2. Causes Involved in the Development of Neurodegenerative Diseases

So far, several reviews have comprehensively conferred the potential health benefits of seaweeds in terms of antioxidant properties. Before summarizing the neuroprotective potentials of seaweeds and their compounds, mechanisms that are involved in the pathogenesis of neurodegenerative diseases are shown through this section to investigate the mechanisms of action of seaweed polyphenols. 

The brain is a demanding organ that utilizes a huge amount of oxygen for optimal function [32]. Several studies have established that the imbalance between pro-oxidant and antioxidant homeostasis, leading to the generation of free radicals, namely, ROS and RNS, may be involved in the pathogenesis of most neurodegenerative disorders [47]. The redox state imbalance is reflected as an increased number of free radicals, ROS, and RNS and a depleted antioxidant defence system, resulting in oxidative stress conditions [48].

The accumulation of ROS and RNS and the interaction between these reactive species can result in lipid peroxidation, protein oxidation, DNA damage, and, ultimately, neuronal cell death [34]. ROSs, such as hydrogen peroxide (H_2_O_2_), superoxide anion (^·^O_2_^−^), and hydroxyl radicals (·OH), and the RNS nitric are formed due to the reduction of oxygen. The members of the ROS group trigger mitochondrial dysfunction and protein misfolding in the endoplasmic reticulum, which lead to ulterior ROS production. Together, these free radicals cause oxidative damage to DNA, lipids, and proteins, resulting in structural changes to the brain, thereby modulating its function, and is eventually followed by a cascade of events that might result in neurodegenerative disease [49].

The loss of certain groups of neurons is a common pathological characteristic of various neurodegenerative diseases [50]. Although cholinergic denervation is recognized as a pathological marker of AD, neuroimaging studies conducted in vivo have revealed the loss of cerebral cholinergic markers in PD as well, with symptoms that are similar or even more severe than those in AD [51]. Consequently, a decline in the levels of acetylcholine (ACh) is seen in both neurodegenerative conditions.

Two types of cholinesterase (ChE) enzymes are present in the CNS: acetylcholinesterase (AChE) and butyrylcholinesterase (BuChE). AChE is an enzyme that specifically targets ACh, breaking it down at cholinergic synapses, whereas BuChE is a non-specific enzyme found in neuroglia and the intestine, liver, kidney, heart, lung, and serum. Both enzymes can split over 10,000 molecules of ACh per second, and therefore, they represent valuable therapeutic targets for the treatment of neurodegenerative diseases [52,53]. ChE inhibitors can slow down the inactivation of ACh post-synaptic release, making them one of the most effective and practical approaches for treating the symptoms of neurodegenerative disorders. Studies have already demonstrated that ChE inhibitors not only increase ACh levels in the brain, but also prevent and reduce the formation of amyloid beta (Aβ) deposits, which protects neurons from neurodegeneration [54].

In AD, Aβ deposits are neurotoxic, triggering the release of pro-inflammatory cytokines, ROS, and RNS, which eventually lead to neuronal dysfunction and cell death [47,48,49]. In PD, the release and accumulation of α-synuclein aggregates activate microglial cells, leading to the production of pro-inflammatory mediators that can also cause neuronal cell death [55].

Antioxidants have been recognized as a successful therapeutic approach for delaying the progression of AD. This is due to the notion that increased ROS levels in the brain are linked to AD development [56]. Due to their high lipid content, high oxygen intake, and weak antioxidant defences, brain cells are particularly vulnerable to free radical damage. Therefore, elevated levels of ROS in brain cells may result in lipid peroxidation, neurodegeneration, and eventually cell death. To avoid this outcome, antioxidants might be helpful for delaying the rapid progression of the disease. 

Studies have shown that natural products derived from plants, animals, algae, and microalgae, including their extracts and bioactive molecules, have therapeutic benefits and neuroprotective action [57].

## 3. Marine Polyphenols Involved in Neuroprotective Activity

### 3.1. Seaweed Polyphenols

**Figure 1:**
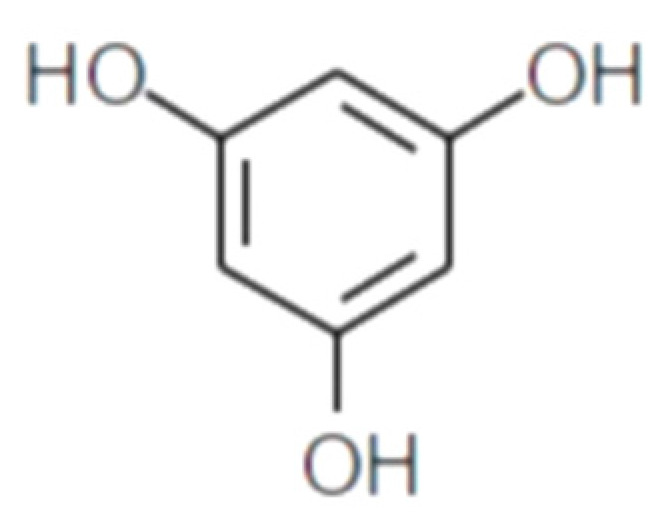
Phloroglucinol (1,3,5-trihydroxybenzene) structure.

**Figure 2:**
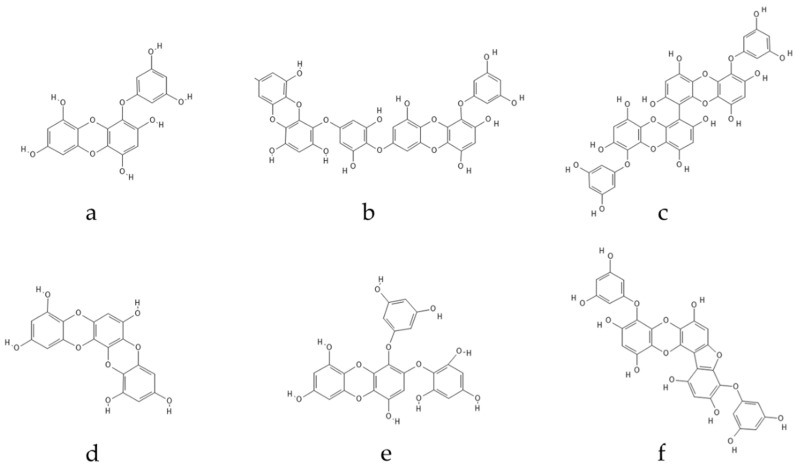
Eckol-class compounds: (**a**) eckol; (**b**) dieckol; (**c**) 6,6-bieckol; (**d**) dioxinodehydroeckol; (**e**) 2-phloroeckol; (**f**) phlorofucofuroeckol.

Polyphenolic secondary metabolites comprise a large collection of chemical compounds found in terrestrial plants [58,59] and seaweeds [60,61]. Tannins, a prevalent group of phenolic metabolites, contain numerous hydroxyl groups and can be classified into three groups. Condensed tannins, which are based on flavonoids, are found predominantly in woody plants, as well as in red wine and tea [62]. Hydrolysable tannins, formed by polyhydric alcohol, where hydroxyl groups are partly or etherified with gallic acid or related compounds, are found in some green algae and are broadly distributed in angiosperms [63]. Phlorotannins, one of several algal polyphenol’s groups, are of great pharmacological significance. They are composed of many phloroglucinol (1,3,5-trihydroxybenzene) (Figure 1) molecules that are linked together in various ways (Figure 2) [64].

Phlorotannins can be split into six distinct groups based on the type of structural connections between phloroglucinol units and the quantity of hydroxyl groups present: phlorethols and fuhalols (phlorotannins with an ether linkage), fucols (which present a phenyl linkage), fucophlorethols (with an ether and phenyl linkage), eckols (with a dibenzodioxin linkage), and carmalols [65]. In the event of cellular damage, these substances are produced via the acetate–malonate pathway [66].

Phlorotannins, a type of polyphenolic compound, are found exclusively in brown algae, and their quantity can vary among species, depending on factors such as algae size, age, tissue type, salinity, season, nutrient levels, intensity of herbivory, light intensity, and water temperature [67]. Similar to other polyphenols, phlorotannins have several remarkable properties relevant to biological systems, including antioxidant [68], anti-inflammatory [69,70], antimicrobial [71], anticancer [72], and antidiabetic [73] activities. Furthermore, phlorotannins play a significant role in neuroprotection via different mechanisms of action.

Although their molecular sizes seem to be important for producing robust interactions with enzymes, variations in the positions and numbers of OH groups and O-bridge linkages may play an even more important role in promoting inhibitory activity. Additionally, phlorotannins can form enzyme–inhibitor complexes by associating with proteins [74].

Reports of different types of phlorotannins in brown seaweeds confirm that the role of these compounds in nature is to protect algae against environmental stressors and predators, although numerous phlorotannins from marine brown algae are known to be an abundant source of nutritious food because of their benefits for health [61,75].

*Ecklonia cava* is a type of marine brown algae that contains a greater abundance of phenolic compounds than other brown algae [76]. Numerous phlorotannins have been isolated from brown seaweeds such as *E. cava*, *Ecklonia kurome*, *Ecklonia bicyclis*, and *Hizikia fusiformis*, and have been found to possess potent antioxidant properties, protecting cells against hydrogen peroxide-induced damage [77,78,79]. Among these phlorotannins, eckol, phlorofucofuroeckol A, dieckol, and 8,8′-bieckol significantly inhibited phospholipid peroxidation at a concentration of 1 M in a liposome system, as well as exhibited effective scavenging activities against superoxide and DPPH (2,2-diphenyl-1-picrylhydrazyl) radicals when compared to ascorbic acid and α-tocopherol [80].

### 3.2. Mechanisms of Action of Antioxidant Seaweed Polyphenols

**Figure 3:**
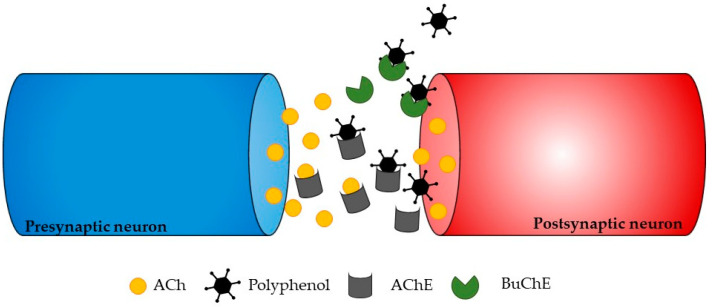
Illustration showing a potential way in which polyphenols may affect neurotransmission. The process of ACh formation occurs briefly before it is broken down by AChE, ultimately leading to the transmission of neurotransmitters to postsynaptic neurons. The inhibition of these enzymes occurs when polyphenols bind to the active sites of AChE or BChE.

ChE inhibitors are a successful approach for treating the symptoms of neurodegenerative disorders, even though various strategies can be used to stop the progression of neurodegeneration. Phlorotannins from *Ecklonia maxima* were isolated by Kannan et al. [81], and the results showed that they had AChE inhibitory action. Dibenzo 1,4-dioxine-2,4,7,9-tetraol and eckol were found to be more effective AChE inhibitors than phloroglucinol. This is likely because they have larger molecules and more hydroxyl groups than phloroglucinol, which can modulate their interactions with AChE and subsequently block it (Figure 3). These findings highlight the potential uses of *E. maxima* as a beneficial ingredient that could be used as additives to foods to act as neuroprotective foods [81]. 

*Ecklonia stolonifera* is a perennial brown alga that is extensively dispersed throughout Korea. Eckol, dieckol, 2-phloroeckol, and 7-phloroeckol were isolated from this alga and showed a selective dose-dependent inhibitory activity against AChE; eckstolonol and phlorofucofurofuroeckol A inhibited both AChE and BuChE. Phloroglucinol and triphlorethol A, a phloroglucinol opened-chain trimer, did not, however, inhibit ChE at the measured concentrations. These results demonstrated that phlorotannins possess structural features that prohibit the binding of substrates to ChE, but they also implied that the degree of polymerization and closed-ring structure must be crucial elements in phlorotannins’ capacity to inhibit ChE [82].

Jung et al. [82] evaluated dieckol isolated from *E. cava*’s neuroprotective benefits by looking at its anti-inflammatory properties. The results showed that dieckol down-regulated nuclear factor b (NF-kB), activated p38 kinase, and/or inhibited ROS signal in microglial cells to significantly inhibit the expression and release of cytokines and mediators that promote inflammation, such as ·NO, PGE2, IL-1β, and TNF-α. The management of reactive stress and neuroinflammation brought on by microglia, which are essential for the beginning of neurodegenerative processes, may therefore be aided by dieckol.

In another work, Yoon et al. [83] isolated phloroglucinol, 6,6′-bieckol and diphlorethohydroxycarmalol (DPHC) from *Ishige okamurae*, a brown edible alga found throughout Korean upper and middle intertidal costal zones. They tested the compound’s ability to inhibit ChE, showing that 6,6′-bieckol and DPHC had strong effects on AChE and modest effects on BuChE, respectively.

Phlorotannins have been shown to have a neuroprotective effect in previous studies through a number of mechanisms, including inhibition of AChE, BuChE, monoamine oxidase, and inhibition of Aβ-precursor protein enzyme 1 (BACE-1) activity [84]. Additionally, phlorotannins have the capacity to modify neuronal receptors and control signalling cascades involved in neuroinflammation, oxidative stress, and neuronal cell death [84]. Although Lee et al. [85] demonstrated that eckol and dieckol were ascribed anti-neuroinflammatory properties in Aβ25–35-treated neuronal PC12 cells, earlier studies showed that eckol, dieckol, and phlorofucofuroeckol A (PFFA) decreased Aβ-induced cell death, inhibited intracellular ROS generation, and increased calcium generation [86].

The most common neurodegenerative condition that causes dementia—a condition marked by increasing memory loss and cognitive decline—in the aging population is AD. The clinical symptoms of AD include the build-up of intracellular neurofibrillary tangles and extracellular A plaques in the brain [87]. Aβ plaques, soluble Aβ oligomers, and protofibrillar forms impair synaptic signalling at neural junctions, interfering with normal neuronal cell function. Their accumulation causes neuronal toxicity [88].

The destruction of Aβ plaques may promote proper neuronal cell activity. Amyloid precursor protein (APP) is first broken down by β-secretase into soluble β-APP fragments (sAPPβ) and the C-terminal β fragment (CTFβ, C99), and then C99 is further broken down by γ-secretase into the APP intracellular domain (AICD) and Aβ. This process is known as the amyloidogenic pathway. Additionally, a number of Aβ peptides form oligomeric clusters and stress neuronal cells via oxidative stress [89].

**Figure 4:**
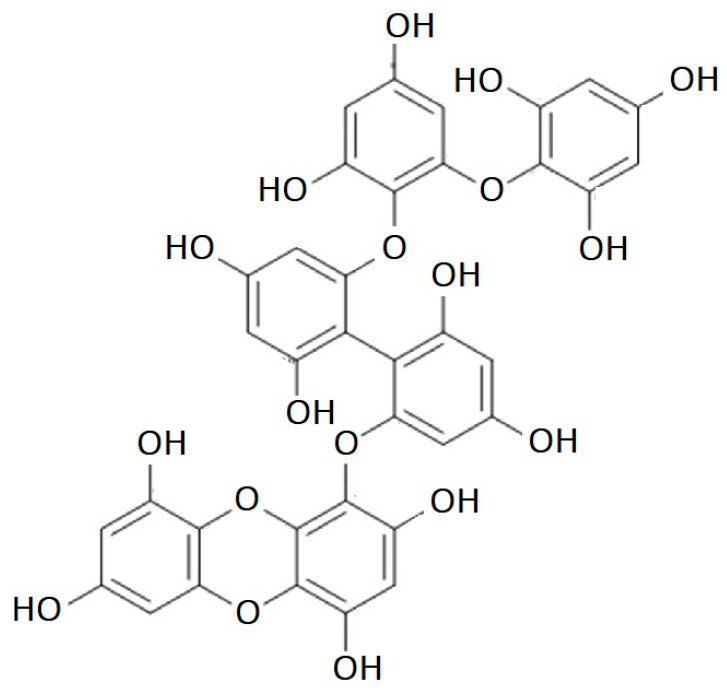
Dibenzodioxin-fucodiphloroethol structure.

Phlorotannins disrupt the amyloidogenic pathway in a variety of ways, decreasing the production of Aβ peptides and lowering the risk of oxidative stress [90]. Recently, Shrestha et al. [91] reported the neuroprotective effects of dibenzodioxin-fucodiphloroethol (Figure 4) that inhibited its neurotoxicity and aggregation of Aβ, providing evidence that phlorotannins have a neuroprotective function through a variety of pathways. 

AChE also engages in non-cholinergic mechanisms, such as accelerating the formation of Aβ plaques through conformational changes in Aβ and raising Aβ toxicity by Aβ-AChE complexes, and it is essential for cholinergic neurotransmission. Therefore, by preventing the accumulation of extracellular Aβ plaques, multi-enzyme target inhibition against AChE may offer a possible therapeutic approach for AD.

Further research is still needed to determine the contributions of phlorotannin classes to neuroprotection activity and their protective mechanisms, such as antioxidant capability and direct modulation of Aβ aggregations.

## 4. Seaweed Polyphenols as Neuroprotective Antioxidants

Throughout this section, in vivo and in vitro investigations conducted in the last ten years are collected (Table 1).

In the study of Shrestha et al. [92], four phlorotannins, namely eckol, dieckol, phlorofucofuroeckol-A (PFFA), and 974-A sourced from the brown seaweed *Ecklonia* species were tested to evaluate their neuroprotective action. Aβ42, H_2_O_2_, and the lipid peroxidant tert-butyl hydroperoxide (t-BHP) were used to induce oxidative stress and toxicity in neuronal PC12 cells. From the results, it was evident that all compounds significantly scavenged ROS. However, only PFFA and 974-A protected PC12 cells from oxidative stress-evoked neurotoxicity, providing significant increases in cell viability in response to both cytosolic (H_2_O_2_) and lipid peroxidation provoked (t-BHP) cell stress. None of the phlorotannins tested inhibited Aβ42 aggregate morphology, which suggested that their neuroprotective activity was unrelated to direct interactions with Aβ42 proteins. Our results indicate that although all phlorotannins tested exhibited ROS scavenging activity, only phlorotannins PFFA and 974-A afforded broader neuroprotective activity in response to both oxidative stress and amyloid beta exposure [92].

When PC12 cells were subjected to t-BHP at concentrations of 150 and 200 µM, cell viability decreased to 63% and 59%, respectively, compared to the control group without t-BHP. However, PFFA and 974-A were found to significantly protect PC12 cells compared to the group treated with t-BHP alone. PFFA increased cell viability to 94% and 92% at 150 and 200 µM of t-BHP, whereas 974-A increased it to 86% and 81% compared to t-BHP alone. Eckol and dieckol, on the other hand, did not exhibit any protective activity against cytosolic oxidative stress induced by t-BHP. PC12 cells pre-treated with phlorotannins and exposed to t-BHP, showed a similar protective effect as in the response to H_2_O_2_. Specifically, the two fucofuroeckols (PFFA and 974-A) were found to significantly protect PC12 cells exposed to 150 and 200 µM of t-BHP, whereas eckol and dieckol did not offer any protection. Intriguingly, all the tested phlorotannins were found to effectively scavenge intracellular ROS, which is likely related to their phenol rings acting as electron traps to scavenge peroxynitrite, superoxide anions, and hydroxyl radicals, as previously noted by the authors [92].

To confirm the scavenging activity of 974-A, it is worth mentioning the investigation of Yotsu-Yamashita et al. [93]. In this work, 974-A and 974-B phlorotannins isolated from *E. kurome* were tested for their antioxidant activity along with phlorofucofuroeckol-A, and dieckol. The text reported the antioxidant activity of these two phlorotannins evaluated using DPPH radical scavenging assay and intracellular radical scavenging assay. The results of the assays suggest that 974-A, 974-B, and dieckol have the strongest intracellular reactive oxygen species reducing capabilities among the tested compounds.

These findings emphasize the potential of a class of phlorotannins from brown seaweed called fucofuroeckols, which can successfully reduce oxidative stress and Aβ-evoked toxicity relevant to neurodegenerative pathways in AD. Additional in vivo studies to further establish preclinical efficacy as a guide to guiding further clinical trials in nutraceutical or pharmaceutical settings are needed in the future, as are studies to differentiate the mechanistic basis for the protection provided by fucofuroeckols [92].

Barbosa and colleagues [94] conducted a study to investigate the potential neuroactive effects of phlorotannin-targeted extracts from various *Fucus* species on human-derived SH-SY5Y cells, focusing on oxidative stress, protein glycation, enzyme inhibition, and cell protection against oxidative glutamate toxicity. The study found that the targeted extracts of *Fucus guiryi* and *Fucus serratus* exhibited significantly higher levels of phlorotannins than *Fucus spiralis* and *Fucus vesiculosus*. Total antioxidant capacity was positively correlated with the phlorotannin content. *F. guiryi* and *F. serratus* extracts showed stronger DPPH scavenging activity, with EC_50_ values of 286.18 and 322.51 μg/mL, respectively, and were also the most effective in reducing lipid peroxidation at EC_50_ concentrations of 845.41 and 932.76 μg/mL, respectively. These activities were found to be strongly correlated with the amount of phlorotannins present in the extracts.

In the study by Barbosa et al. [94], phlorotannin-targeted extracts from various *Fucus* species, as well as phloroglucinol, a constituent unit of phlorotannins, were found to inhibit the formation of fluorescent advanced glycation end products (AGEs) in a concentration-dependent manner. AGEs are associated with the pathology of AD, as they induce neurodegeneration by interacting with the receptor for AGE (RAGE). *F. guiryi* and *F. serratus* extracts were more effective than those from *F. spiralis* and *F. vesiculosus* in inhibiting protein glycation, with a strong correlation observed between the total phlorotannin content and the capacity to inhibit the formation of AGEs. The study also found that the most potent extracts, *F. guiryi* and *F. serratus*, contained primarily low-molecular-weight phlorotannins (370–498 Da) and had a low degree of polymerization, indicating the significance of the qualitative composition of the extracts in addition to the phlorotannin content.

The accumulation of neurotoxic aggregated Aβ peptides is believed to play a crucial role in the pathogenesis and neurological damage of AD. Currently, there is no effective medication available to treat or prevent AD, and the only available option is to improve the quality of life for patients with the disease [95]. In the search for new pharmacotherapies, one approach is to target the aggregation and toxicity of Aβ [96]. Therefore, there is a growing interest in the commercial sector to identify compounds from novel sources that can disrupt Aβ aggregation or protect neuronal cells from Aβ toxicity, with the goal of developing potential disease-modifying therapeutics for the treatment and prevention of AD [97].

This study aimed to investigate the neuroprotective effects of different extracts of *Ecklonia radiata* on Aβ42 toxicity in PC12 cells. The study also aimed to identify major phlorotannins in the extracts associated with neuroprotection using high-performance centrifugal partitioning chromatography (HPCPC) and LC-MS/MS. The ethyl acetate (EA) fraction containing 62% phlorotannins demonstrated the most effective neuroprotective activity against Aβ42 toxicity, inhibiting neurotoxicity at all Aβ42 concentrations. The fraction also showed a significant reduction in Aβ aggregate density but did not affect overall aggregate morphology. The study used centrifugal partitioning chromatography to isolate the major component, eckol, in high yield and liquid chromatography–mass spectrometry to characterize the major components of the EA fraction. The results suggested that the presence of eckol-type phlorotannins is associated with the neuroprotective bioactivity of *E. radiata*, which may have potential nutraceutical and biopharmaceutical uses in treating dementia [98].

The extracts from *E. radiata* were found to be non-toxic to PC12 cells at concentrations up to 100 µg/mL. When PC12 cells were incubated with Aβ42 (0.05–1 µM) for 48 h, there was a concentration-dependent decrease in cell viability, with up to 79% reduction observed at 1 µM Aβ42. However, all the extracts tested demonstrated protection against Aβ-induced PC12 neuronal cell toxicity. The ethanolic extract increased cell viability to over 89% compared to the control group (79%), consistent with a previous study. Of note, the EA fraction exhibited the most significant protective activity across all Aβ concentrations, increasing cell viability to more than 100% compared to the control at low concentrations of Aβ, indicating a potential proliferative or stimulating effect of EA on mitochondrial activity. Overall, the EA extract showed significant protection against neurotoxicity in PC12 cells at most Aβ concentrations, which was associated with a reduction in both the prevalence and density of Aβ42 aggregates. These findings suggest that *E. radiata* may be a promising natural source of therapeutic neuroprotective phlorotannins; however, further studies are needed to identify other novel compounds associated with *Ecklonia* species [98].

Alghazwi et al. [97] conducted a study to investigate the impact of extract composition on the neuroprotective activities of *E. radiata*. The study used six fractions of *E. radiata* extract, including crude extract (CE), phlorotannin (PT), polysaccharide (PS), free sugar (FS), low molecular weight (LM), and high molecular weight (HM), to evaluate their effects against Aβ42 and oxidative stress in the PC12 neuronal cell line. The results showed that all fractions inhibited apoptosis induced by Aβ42 and enhanced neurite outgrowth activity, indicating the potential of using *E. radiata* for improving neuroprotective activities. Moreover, all fractions demonstrated concentration-dependent neuroprotective activity against Aβ42-induced cytotoxicity, with the CE, PT, and PS fractions showing the highest neuroprotective activity, increasing cell viability to more than 92% at a concentration ranging from 50 to 100 μg/mL. Importantly, the MTT assay revealed that three fractions (PT, FS, and LM) did not exhibit significant cytotoxicity to PC12 cells, even at the highest concentration of 100 μg/mL, indicating the safety of using *E. radiata* fractions as potential functional food or dietary supplements to support neurological function [97].

Alghazwi and colleagues investigated the efficacy of different fractions of *E. radiata* against hydrogen peroxide-induced neurotoxicity in PC12 cells. A concentration of 100 μM hydrogen peroxide reduced cell viability by 38% compared to PBS control. However, all fractions except for HM demonstrated neuroprotective activity by reducing neurotoxicity. The CE and PS fractions showed significant antioxidant activity at 12.5 μg/mL, whereas the PT, FS, and LM fractions showed antioxidant activity at 25 μg/mL. Among these fractions, CE exhibited the highest antioxidant activity, recovering cell viability from 62% to 84% at a concentration of 50 μg/mL, whereas LM showed the lowest activity, with 100 μg/mL resulting in a recovery from 62% to 75% cell viability. The remaining three fractions (PS, PT, and FS) demonstrated varying degrees of protection, with a recovery of 63–82% viability at tested concentrations ranging from 3.125 to 100 μg/mL [97].

Alghazwi et al. [99] found that the PT fraction had the strongest anti-apoptotic activity, with an apoptosis rate of less than 7%. This could be attributed to the high degree of hydroxylation in phlorotannin compounds, as a previous study demonstrated the neuroprotective effects of 6,6′-bieckol against high glucose-induced cytotoxicity in INS-1 cells. These results suggest that further research should investigate the neuroprotective compounds in *E. radiata* as potential functional food supplements for managing neurodegenerative disorders such as AD. It is recommended that additional studies be conducted to identify the specific components responsible for the neuroprotective activities of seaweed extracts, including polysaccharides and phlorotannins.

In a study conducted by Alghazwi et al. [100], the neuroprotective effects of phlorotannin-rich fucoidan samples from *F. vesiculosus* and fucoidans from *Undaria pinnatifida* were observed due to their inhibitory effect on Aβ aggregation and Aβ42-induced cytotoxicity in PC12 cells. Five types of fucoidan samples (FE, FF, and S from *F. vesiculosus* and UE and UF from *U. pinnatifida*) were tested for their neuroprotective potential. The fucoidan S sample showed the lowest neuroprotective activity, where cell viability did not exceed 90% even at the highest concentration. On the other hand, fucoidan UE demonstrated higher activity than fucoidan UF, although it is not known which structural aspect is behind this finding. Fucoidan samples FE and FF from *F. vesiculosus* inhibited cytotoxicity induced by Aβ42, with cell viability exceeding 80% at the lowest concentration (3.125 μg/mL), whereas at the highest concentration (100 μg/mL), it reached <98%. Fucoidan S from *F. vesiculosus* had the greatest effect on preventing Aβ42 clustering. Fucoidan from *U. pinnatifida* extract, on the other hand, demonstrated a stronger anti-aggregation impact against Aβ42. The sample S from *F. vesiculosus*, rich in polyphenols showed the greatest anti-Aβ42 aggregation effects compared to other samples, possibly due to the presence of phlorotannin, the most common polyphenolic compound in brown algae [60,61]. The findings suggest that fucoidan neuroprotective activity depends on the structure of the polysaccharides, which can differ depending on the source and the techniques used to purify it. Fucoidan samples might neutralize free radicals to provide an antioxidant defence [101]. These bioactive substances that inhibit Aβ aggregation may offer alternative treatment for AD patients [100].

**Figure 5:**
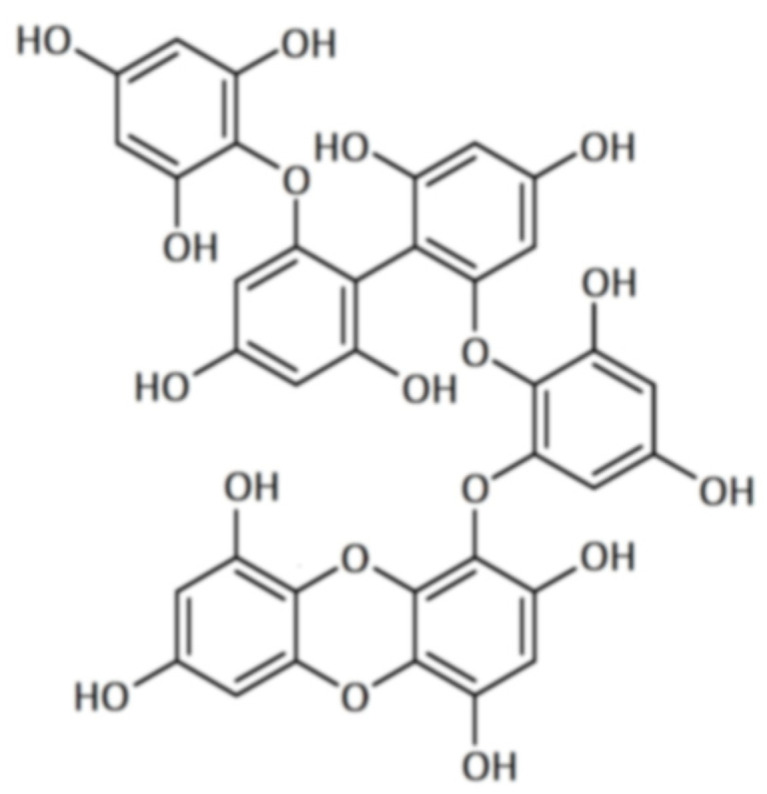
Eckmaxol structure.

Another unique phlorotannin (eckmaxol, Figure 5) isolated from *Ecklonia maxima*, also exhibited anti-amyloidogenic activity [102].

Eckmaxol is a phlorotannin that was extracted from *E. maxima* and has 13 aromatic hydroxy groups in its structure, a feature that suggests it may exhibit potent antioxidative stress effects. It was found that eckmaxol prevented Aβ oligomer-induced cell death and cytotoxicity in neuroblastoma cell line SH-SY5Y in a concentration-dependent manner. At a concentration of about 20 μM, eckmaxol exhibited its highest level of neuroprotective activity (73.08 ± 5.74%) without affecting the survival of SH-SY5Y cells. The MTT assay demonstrated that longer treatment times greatly increased the neuroprotective effects of eckmaxol. According to the results, eckmaxol protected SH-SY5Y cells from apoptosis brought on by Aβ oligomers.

It was also found that after 1 h treatment with 20 μM eckmaxol, the Aβ oligomer-induced increase in the intracellular ROS level significantly reduced from 154.92 ± 22.54% to 70.42 ± 3.28%, suggesting that this phlorotannin can attenuate Aβ oligomer-induced oxidative stress in SH-SY5Y cells. The addition of eckmaxol alone at 20 μM did not increase intracellular ROS (98.69 ± 2.32%) in SH-SY5Y cells. Therefore, these results suggested that eckmaxol demonstrates potent neuroprotective effects [102]. 

The recent study of Meshalkina et al. [103] assessed the anti-neurodegenerative activity of phlorotannin extracted from *F. vesiculosus* and *Pelvetia canaliculata* for differentiated SH-SY5Y cells. The extracts of both brown algae showed modest but significant protective effects in the paraquat cell model of PD. Specifically, the intracellular phlorotannins of *F. vesiculosus* significantly improved the viability of paraquat-treated cells only at the highest concentration tested (10 µg/mL), which was toxic to the control cells not treated with paraquat, whereas the extract of *P. canaliculata* demonstrated a protective effect at all the concentrations tested. In the Aβ25–35 cell model of AD, tested extracts of brown algae demonstrated well-pronounced protective activity, noticed by cell viability that could be restored to almost the control levels. The solutions containing the extracts were applied to the cells for 0.5 h prior to the solution of Aβ25–35, and the resulting signal was compared with the control of Aβ25–35-only wells. In these experiments, the extracts of *P. canaliculata* demonstrated more pronounced protective activities at all the concentrations, whiwhereasle extracts of *F. vesiculosus* exhibited protective effect only at the highest concentrations of 5 µg/mL and 10 µg/mL. However, the highest concentrations in the tested range produced the strongest effect, implying therapeutic doses of the extracts may be rather close to the toxic doses.

Yang et al. [104] investigated the action of phloroglucinol (1,3,5–trihydroxybenzene), a component of phlorotannins extracted from *E. cava* to verify the occurrence of therapeutic activities in AD. Phloroglucinol attenuates the increase in ROS accumulation induced by oligomeric Aβ42 treatment in HT-22, hippocampal cell line. In addition, phloroglucinol was shown to ameliorate the reduction in dendritic spine density induced by Aβ42 treatment in vivo in rat primary hippocampal neuron cultures. The results showed that administration of phloroglucinol to the hippocampal region attenuated the impairments in cognitive dysfunction observed in vivo in 22-week-old 5XFAD (Tg6799) mice. These results indicate that phloroglucinol displays therapeutic potential for AD by reducing the cellular ROS levels [104].

In the experiment, cells were treated with 10 μg/mL phloroglucinol for an hour before being treated with 8 μM Aβ42 for six hours. Fluorescence intensity showed that phloroglucinol pre-treatment significantly reduced ROS accumulation caused by Aβ42, indicating that phloroglucinol can lower ROS accumulation caused by Aβ42 [104]. After three days, the mice underwent the Morris water maze test to assess their spatial learning and memory capacities. The AD Tg mouse model showed longer latency in locating the hidden platform compared to the wild-type (WT) mice. However, Tg mice treated with phloroglucinol showed a significantly shorter escape latency than Tg mice given the vehicle on the fourth day of the learning stage. The probe test showed no significant differences in the time spent in the quadrant between the groups. The T-maze test, which evaluated working memory, was performed on the eleventh day after injection. The Tg mice exhibited decreased spontaneous alternation ratio compared to WT mice, but the administration of phloroglucinol restored this ratio significantly. There were no significant differences in Aβ level or the level of neprilysin, a major Aβ degrading enzyme, between the groups, suggesting that phloroglucinol did not affect Aβ generation or degradation [104].

Data revealed that phloroglucinol exhibits therapeutic potential in AD and may postpone the disease progression by reducing cognitive function deficits by working as an antioxidant. Based on these findings, the authors hypothesize that phloroglucinol’s beneficial effects on the behavioural phenotype of AD are not caused by a direct impact on the production and/or degradation of Aβ, but rather by protective effects against a decrease in the density of dendritic spines and synaptic proteins such as synaptophysin and PSD-95. Antioxidant therapy may therefore be a strategy that addresses numerous molecular processes thought to be involved in the pathogenesis of AD [104].

The recent study of Myung et al. [105] shows that dieckol and phlorofucofuroeckol (PFF) phlorotannins present in *E. cava* are involved in the inhibition of acetylcholinesterase, and therefore, they may improve cognitive functions, suggesting that they have the potential ability to enhance memory in several neurodegenerative disorders. Dieckol and phlorofucofuroeckol were administered orally to ethanol pre-treated mice. The repeated administration of either dieckol or PFF in a dose-dependent manner reduced the inhibition of latency by the administration of ethanol. Major central neurotransmitter levels were assessed in the striatum, hippocampus, and frontal cortex of the mouse brain to determine the mechanism of memory-enhancing actions. Levels of some neurotransmitters that were affected by the ethanol treatment changed due to the action of dieckol and PFF. It is noteworthy that both dieckol and PFF increased the level of acetylcholine, and they exerted anticholinesterase activities. Overall, the memory-enhancing abilities of dieckol and PFF may result from, at least in part, an increase in the brain acetylcholine levels by inhibiting acetylcholinesterase [105].

Yang et al. [106] conducted a recent study that showed the effectiveness of oral administration of phloroglucinol from *E. cava* for improving cognitive impairments in 6-month-old 5X familial AD (5XFAD) mice. The treatment resulted in a decrease in the number of amyloid plaques and the protein level of BACE1, a major enzyme involved in amyloid precursor protein cleavage, along with γ-secretase. Moreover, phloroglucinol restored the decrease in dendritic spine density and the number of mature spines in the hippocampi of 5XFAD mice. These findings suggest that phloroglucinol has therapeutic potential for treating AD by mitigating neuropathological symptoms and behavioural phenotypes in the 5XFAD mouse model [106].

The studies presented in this passage highlight the potential therapeutic effects of phlorotannins extracted from *E. cava* in reducing cognitive deficits associated with AD. Although the results obtained from cell and animal models are promising, it is important to note that these models have limitations and differences in their relevance to human biology. Cell cultures provide a controlled environment for studying the effects of compounds on specific cellular pathways. However, they lack the complex interactions between different cell types and organ systems present in vivo, which may affect the effectiveness and safety of therapeutic interventions. Animal models can provide a more comprehensive understanding of the effects of a treatment in vivo, but there are variations between species and strains, and the pathology and progression of diseases may differ from those in humans. Additionally, the experiments described in this passage focus on specific aspects of AD pathology, such as oxidative stress, amyloid plaque accumulation, and cholinergic deficits. AD is a multifactorial disease with complex underlying mechanisms, and the effects of phlorotannins on other aspects of the disease, such as neuroinflammation, remain to be studied. Overall, although the studies presented in this passage suggest that phlorotannins from *E. cava* may have therapeutic potential for AD, further studies are needed to fully understand the efficacy and safety of this treatment as well as its potential limitations and differences in relevance to human biology.

Though there is growing interest in the potential neuroprotective effects of marine macroalgae polyphenols, it is important to acknowledge the limitations of the current studies. Most studies have been conducted in vitro or in animal models, and the translation of these findings to human clinical trials remains uncertain. Additionally, most studies have focused on the antioxidant properties of marine polyphenols, and further research is needed to fully understand the mechanisms underlying their neuroprotective effects. Furthermore, the bioavailability and pharmacokinetics of marine polyphenols need to be studied to determine optimal dosing and delivery methods. These limitations highlight the need for further research to fully realize the potential of marine macroalgae polyphenols as neuroprotective agents.

**Table 1:** In vitro and in vivo assays performed to investigate seaweed polyphenols neuroprotective activity.

Species	Extracted Compound	Exhibited Effect	In Vitro/ In Vivo Assay	References
*Ecklonia cava*	Dieckol Phlorofucofuroeckol	Inhibition of AChE. Potential ability to enhance memory in neurodegenerative disorders	In vivo (Ethanol pre-treated mice)	[105]
*Ecklonia cava*	Phloroglucinol	Limited increase in ROS accumulation	In vitro (SH-SY5Y cells)	[104]
		reduction in dendritic spine density in mice	In vivo (5XFAD mice)	
*Ecklonia cava*	Phloroglucinol	Improvement of cognitive impairments	In vivo (5XFAD mice)	[106]
*Ecklonia kurome*	Dieckol phlorofucofuroeckol-A 974-A 974-B	All compounds showed ROS scavenging activity	In vitro (DCFH-DA assay)	[93]
*Ecklonia maxima*	Eckmaxol	Neuroprotective activity prevention aggregation of β-amyloid	In vitro (SH-SY5Y cells)	[102]
*Ecklonia radiata*	Eckol-type phlorotannins	Neuroprotective activity	In vitro (PC12 cells)	[98]
*Ecklonia radiata*	Phlorotannin	Apoptosis inhibition. Neuroprotective activity	In vitro (PC12 cells)	[97]
*Ecklonia* sp.	Eckol Dieckol Phlorofucofuroeckol-A 974-A	All compounds showed ROS scavenging activity. Phlorofucofuroeckol-A and 974-A showed neuroprotective activity	In vitro (PC12 cells)	[92]
*Fucus guiryi* *Fucus serratus* *Fucus spiralis* *Fucus vesiculosus*	Phlorotannin	All compounds showed ROS scavenging activity (stronger activity for *Fucus guiryi* and *Fucus serratus* extracts)	In vitro (SH-SY5Y cells)	[94]
*Fucus vesiculosus* *Undaria pinnatifida*	Phlorotannin-rich fucoidan Fucoidan	Neuroprotective activity. prevention aggregation of β-amyloid	In vitro (PC12 cells)	[100]
*Fucus vesiculosus Pelvetia canaliculata*	Phlorotannin	Neuroprotective activity	In vitro (SH-SY5Y cells)	[105]

## 5. Conclusions

Seaweeds are enriched with several compounds with therapeutic potential. Among them, polyphenols have clearly exhibited interesting in vitro and in vivo pharmacological properties related with antioxidant properties. Data collected in the present review suggest that seaweed can be important sources of polyphenolic compounds with potential applications as pharmaceutical or nutraceutical agents for prevention and control of neurodegenerative processes through different pathways. The manuscript presents a comprehensive review of the neuroprotective potential of polyphenols from seaweeds. Although some previous studies have focused on the neuroprotective effects of seaweed-derived compounds, this review aims to synthesize and critically evaluate the current state of knowledge on this topic. The manuscript builds on previous research by providing a more in-depth and up-to-date analysis of the neuroprotective properties of marine polyphenols, particularly in relation to neurodegenerative diseases. This review also highlights the potential of these compounds as a source of natural and innovative neuroprotective drugs, given their antioxidant and signaling pathway-altering properties. Furthermore, this review presents an extensive evaluation of preclinical and clinical studies investigating the neuroprotective effects of seaweed-derived compounds. This comprehensive analysis has the potential to aid in the identification of new therapeutic strategies for the treatment of neurodegenerative diseases, which currently lack effective treatments. Overall, the manuscript’s contribution lies in its synthesis and evaluation of recent literature as well as its potential to provide new insights and strategies for the development of neuroprotective drugs derived from seaweeds polyphenols, aiding patients that suffer from neurological diseases to achieve a better life quality.

## Data Availability

Not applicable.
